# Osteoblastoma of scaphoid of the carpus: a case report

**DOI:** 10.4314/pamj.v8i1.71154

**Published:** 2011-03-14

**Authors:** Hanane Bahouq, Fedoua Allali, Najia Hajjaj-Hassouni

**Affiliations:** 1 Department of rheumatology, CHU Ibn Sina, Hospital El Ayachi, Rabat - Sale, Morocco

**Keywords:** Osteoblastoma, scaphoid, hand, proximal row carpectomy, Morocco

## Abstract

Osteoblastoma is a rather uncommon tumor, especially when located in the wrist (and hand). Its diagnosis therefore appears to be problematic. We describe a case of osteoblastoma of the scaphoid bone and its management. To our knowledge, this is the fourth case of osteoblatoma of the carpus reported in the literature. Proximal row carpectomy was chosen because of the aggressive nature of the tumor. A tendon transfer was performed in the same session for wrist stabilization. Other authors have opted for a more conservative approach (tumor curettage with bone grafting).

## Introduction

Osteoblastoma is a rare primary neoplasm of the bone and is categorized as a benign tumor. However, aggressive types of osteoblastoma have been described with characteristics similar to those of osteosarcoma [1].

Osteoblastomas account for only 0.5 to 2% of all primary bone tumors and for only 3% of benign bone tumors [2]. The usual location is the pelvis, spine and long bones; very rarely the tumor presents in the hand; its localization in the scaphoid carpal bone is exceptional; only three cases have been reported in the literature so far [3]. The rarity of this location has prompted the report of this case.

## Case report

**Figure 1:**
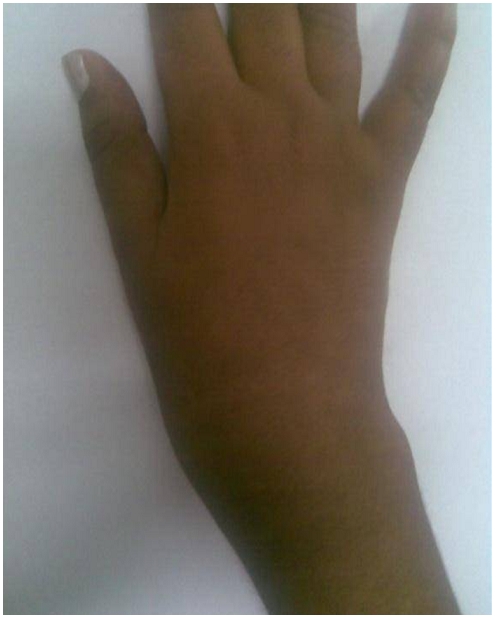
Front view of a tumefaction of the right wrist in a Moroccan patient with an osteoblastoma of scaphoid of the carpus

A 17 years old, right handed woman presented with a 7 months history of progressive right wrist pain which was not relieved by conservative treatment. There was no history of trauma. Examination revealed a painful swelling over the radial aspect of the right wrist (Figure 1). Movements were slightly restricted by pain. Extension of the hand was reduced to 15°, flexion to 20°.

**Figure 2:**
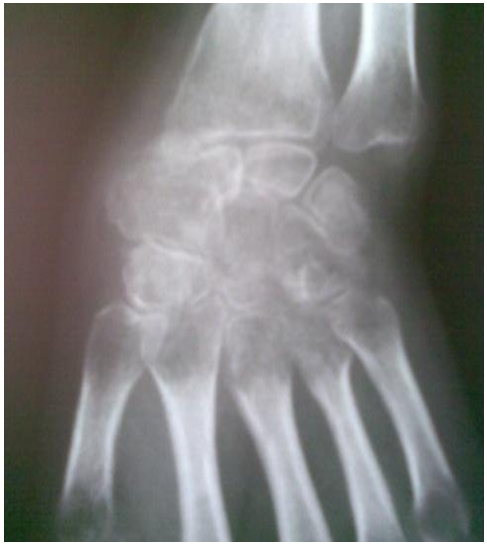
X-ray of the right hand of a Moroccan patient with an osteoblastoma of scaphoid of the carpus showing a demineralization of the scaphoid

**Figure 3:**
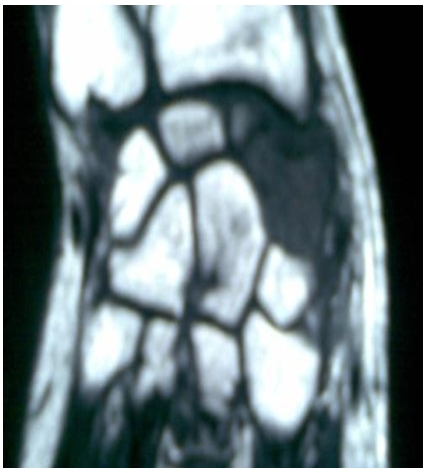
T1-weighted sagittal magnetic resonance image. This image demonstrates a focal lesion of low signal intensity in the carpal scaphoid, with surrounding low-signal-intensity edema in a Moroccan patient with an osteoblastoma of scaphoid of the carpus

**Figure 4:**
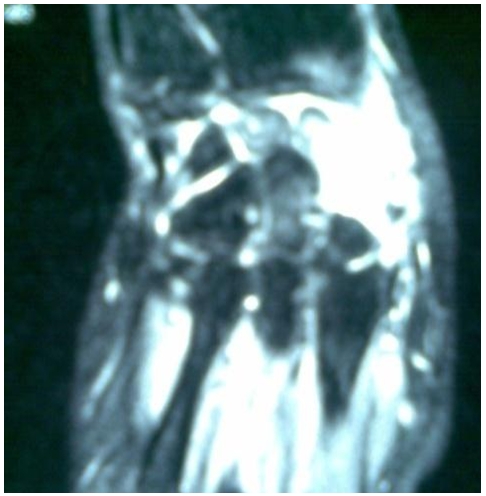
Focal lesion in the carpal scaphoid in a Moroccan patient with an osteoblastoma of scaphoid of the carpus. Surrounding the lesion is a low-signal-intensity edema rose by gadolinium, discreetly heterogeneous

Hand X-ray revealed the demineralization of a giant scaphoid (Figure 2). T1 weight led coronal MR images before and after administration of gadolinium showed an intraosseous mass, with partial erosion of the scaphoid; coupled to homogeneous low signal intensity on the T1 weighted image and high signal intensity on the T2 weighted with heterogeneous signal (figure 3, 4).

A biopsy was then performed showing, macroscopically, a cavity containing a spongy bone and a reddish soft fabric. Microscopy revealed a proliferation of well differentiated osteoblasts, numerous giant cells and a moderately developed conjonctivo-vascular fabric. The diagnosis of osteoblastoma was then retained.

Synovectomy of the extensors; associated to the excision of the posterior capsule and the mass was performed; allowing for complete exposure of the carpus. In view of the aggressiveness of the lesion, complete excision of the proximal carpal row was performed (including the pisiform), followed by a thorough synovectomy and removal of the yellowish soft tissue mass infiltrations. As the capsulectomy-carpectomy had rendered the wrist completely unstable, a tendon transfer using the extensor carpi radialis brevis was performed to provide stability. The wrist was finally immobilized in a short arm plaster cast for six weeks.

Histologic examination of the scaphoid showed a lesion consisting mainly of a benign osteoblastic proliferation. A large osteoblastic nidus was seen, formed by irregular trabeculae of osteoid that were encircled by numerous osteoblasts with dystrophic nuclei. A large number of osteoclasts were also present. There were no suspected signs of malignancy, especially osteosarcoma. The final diagnosis of osteoblastoma was retained.

One week postoperatively, the patient was practically pain-free. After removal of the plaster a rehabilitation program was started. Three months postoperatively her progress was already very satisfactory; the wrist was completely pain-free and stable. The range of movement was: dorsiflexion 25°, palmar flexion 20°, ulnar deviation 15°, radial deviation 10°, with full supination and pronation. The treatment continued with further physiotherapy and intermittent splinting. Due to the possibilities of recurrence the patient was advised on the need of a long-term follow-up.

## Discussion

This observation highlights the difficulties associated with the diagnosis and management of osteoblastoma in this unusual location. Bones of the carpus are rare locations for osteoblastoma [4]; three cases were published separately by Ragois [5], Xarchas [3] and Fanning [5]. The series of osteoblastoma of Lichtenstein and Mangini [6] does not give a report on this localization on the scaphoid. Other reported sites included the bones of the feet and ankles; the skull and facial bones; the ribs; the spine and the sternum, clavicles, scapulae, patellae, and pelvis [7-10]. Osteoblastoma affects males more often than females, with an incidence of 2-3:1 [2].

Although osteoblastoma can occur at any age, the tumor predominantly affects younger persons, with about 80% of these tumors occurring before 30 years [2].

Osteoblastoma as a separate entity was first described by Jaffee in 1932 [4]. This was a case of osteoblastoma of a metacarpal. In 1978 Mosher et al. described a case of osteoblastoma of the fifth metacarpal in a 10-year-old boy [2]. In 1993 Fanning and Lucas [11] reported a case of osteoblastoma of the scaphoid in a 31-year-old woman with a long-standing history of wrist pain. The authors performed curettage of the tumor and cancellous bone grafting of the scaphoïd [12]. Three years later there was no recurrence, but the patient still had pain and stiffness. A second case of scaphoïd bone osteoblastoma was reported by Ragois et al. in 2000 [5]. The authors stressed the rarity of the tumor, the difficulties in its diagnosis and tits local aggressiveness that led them to a scaphoidectomy [3, 5].

Preliminary radiologic workup should consist of radiography at the site of the pain. However, computed tomography (CT) scanning is often necessary to support clinical and plain radiographic findings that are suggestive of osteoblastoma. CT scan also plays an important role in better defining the margins of the lesion for potential surgery. CT scans are best used to further characterize the lesion with regard to the presence of a nidus and matrix mineralization [13,14].

Although osteoblastomas demonstrate an increased radiotracer accumulation on bone scans, the tumors´ appearance is nonspecific, and it is difficult to differentiate these lesions from those due to other etiologies that involve increased radiotracer accumulation in the bone.

Similar to CT scans, MRIs can help in defining the extent of the osteoblastoma. MRI is often superior to CT scanning with regard to the detection of a soft-tissue mass, although this is a relatively uncommon feature of osteoblastomas. A typical osteoblastoma has decreased signal intensity on T1-weighted images; the signal intensity of osteoblastomas on T2-weighted images is variable. Although, in general, the tumors are hyperintense relative to marrow on T2-weighted images, many osteoblastomas encountered have been heterogeneously hypointense relative to marrow on nonfat-suppressed T2-weighted images, presumably reflecting the ossific matrix of the lesion [15].

MRI defines soft-tissue extension, although the finding is not typical of osteoblastoma [16,17]. Osteoblastomas accumulate radionuclide on bone scintigraphy studies, however, scintigraphic appearance of the tumors is nonspecific [15].

When clinical and radiographic findings are consistent with the diagnosis of osteoblastoma, an excisional biopsy is appropriate [18]. Percutaneous CT-guided biopsy is an outpatient nonsurgical procedure with minimal complications. Although it resembles osteoid osteoma there are significant differences between the two lesions in their clinical, radiological and histological appearance [18]. Pain is more prominent in osteoid osteoma as is the characteristic radiolucent nidus [2,3,6,18,19] with typical radiographic features of a nidus and a surrounding area of reactive bone. The nidus of an osteoblastoma is larger than that of an osteoid osteoma, with some investigators using 2 cm as a size distinction. Histologically, osteoblastomas are similar to osteoid osteomas, both producing osteoid and primitive woven bone amidst fibrovascular connective tissue. Although the tumor is usually considered benign, a controversial aggressive variant has been described in the literature, with histologic features similar to those of malignant tumors such as osteosarcomas [1,5,20]. Differential diagnosis of scaphoïd bone tumors should also include intraosseous ganglion cysts, aneurismal bone cyst, enchondroma, giant cell tumor and osteosarcoma. These lesions have been found in all carpal bones [3].

The assessment of the proliferative antigen expression yields predictive indicators in soft tissues and bone tumors, with several studies demonstrating the clinical value of anti-PCNA (proliferating cell nuclear antigen) and anti-p53 monoclonal antibodies in these tumors. Rossner and al [21,22] reported mutation of the p53 gene at exon 5 in a patient with malignant osteoblastoma. Radig and al [21,22] described another mutation at exon 7 in a patient with osteoblastoma-like osteosarcoma. Luca and al [21,22] have previously reported abnormalities in exon 10 of the p53 gene of osteosarcomas, suggesting that exon 10, which is probably involved in the protein oligomerization, is essential for protein function.

Rearrangements involving multiple chromosomes (2, 5, 6, 8, 9, 11, 12, 13, 14, 16, 18, 19, 21, and X) [22] and balanced translocations involving chromosomes 4, 7, and 14 have been reported in the aggressive variant of osteoblastoma. Osteoblastomas can be difficult to treat. Curettage and grafting are performed with most active lesions. A bloc resection can also be performed. Larger lesions may require internal fixation. Irradiation is another therapeutic option [23,24]. The overall recurrence rate is approximately 20%; therefore, close follow-up monitoring of patients is required. Rates of recurrence are lower with wide surgical excision, but the location of the lesion does not always allow for this option. Cementation in conjunction with excision may be helpful in extending the surgical margin.

In our case the tumor of the scaphoïd appeared to be even more aggressive and although localized, still very destructive. This aggressive nature of osteoblastoma has already been described, as well as the possibilities of local recurrence or malignant transformation into osteosarcoma [1, 3, 5].

Locally aggressive behavior has been noted in some osteoblastomas; however, malignant transformation of the tumors is rare. Approximately 25% of patients have radiographic and histologic aspects that may suggest malignancy, such as large epithelioid osteoblasts, bizarre cells, mitosis, and lace- or sheet-like asteroid production. The term aggressive osteoblastoma was considered preferable because it designates a tumor with local destructive behavior, but without metastatic potential [2].

We believe that wide resection (with long-term follow-up) should be the treatment of choice in cases of aggressive osteoblastoma [12,23]. In our case the extensive invasion of the soft tissues justified complete resection of the dorsal capsule and ligaments. This in turn created the need for wrist stabilization with a double tendon transfer [3]. Transfer of the radial wrist extensors gave a reasonably stable and balanced wrist, and we therefore recommend the technique in similar cases.

## Conclusion

With four reported cases, we believe that osteoblastoma of the scaphoid should be recognized as an uncommon but still possible cause of wrist pain. Its differential diagnosis mainly includes osteoid osteoma intraosseous ganglion cysts and osteosarcoma. Cytogenetic analysis of the osteoblastoma revealed a diploid clone exhibiting a complex translocation. Additional studies are required to address the significance of these abnormalities and to clarify their role in the pathogenesis of a lesion with uncertain potential. When diagnosed, osteoblastoma of the scaphoid should be treated with extreme care due to its potentially aggressive nature.

## Competing interests

The authors declare no competing interests

## Authors’ contribution

All authors have read and agreed to the final version of this manuscript and have equally contributed to its content and to the management of the case.
